# Revising Endosomal Trafficking under Insulin Receptor Activation

**DOI:** 10.3390/ijms22136978

**Published:** 2021-06-29

**Authors:** Maria J. Iraburu, Tommy Garner, Cristina Montiel-Duarte

**Affiliations:** 1 Department of Biochemistry and Genetics, University of Navarra, 31008 Pamplona, Spain; miraburu@unav.es; 2 The John van Geest Cancer Research Centre, School of Science and Technology, Nottingham Trent University, Nottingham NG11 8NS, UK; tommy.garner2017@my.ntu.ac.uk

**Keywords:** insulin receptor, endocytosis, receptor trafficking, endosomal recycling compartment, Arf GTPases, Ras GTPases

## Abstract

The endocytosis of ligand-bound receptors and their eventual recycling to the plasma membrane (PM) are processes that have an influence on signalling activity and therefore on many cell functions, including migration and proliferation. Like other tyrosine kinase receptors (TKR), the insulin receptor (INSR) has been shown to be endocytosed by clathrin-dependent and -independent mechanisms. Once at the early endosome (EE), the sorting of the receptor, either to the late endosome (LE) for degradation or back to the PM through slow or fast recycling pathways, will determine the intensity and duration of insulin effects. Both the endocytic and the endosomic pathways are regulated by many proteins, the Arf and Rab families of small GTPases being some of the most relevant. Here, we argue for a specific role for the slow recycling route, whilst we review the main molecular mechanisms involved in INSR endocytosis, sorting and recycling, as well as their possible role in cell functions.

## 1. Introduction

Endocytosis, a process which initiates with the invagination of the plasma membrane (PM) and the formation of vesicles, allows eukaryotic cells to internalize a wide range of materials including nutrients, extracellular matrix (ECM) components, solutes, membrane proteins, antibodies and receptor-ligand complexes. Endosomes are the vesicles responsible for the control of incoming cargos, sorting them to different destinations to be processed, recycled back to the surface or degraded for elimination [1]. In this way, endosomes promote a fine balance between the exiting and entering of materials in the cell and consequently maintain cell homeostasis. The endocytic system is connected with exosomic vesicles and therefore with the endoplasmic reticulum (ER) and Golgi apparatus, organelles responsible for the processing and export of proteins. In fact, some endosomes, ER and vesicles from the trans-Golgi system are localised around the nucleus, forming the so-called perinuclear cloud, whose dynamics are still poorly understood [1,2]. Many proteins belonging to the small GTPase superfamily, such as members of the ADP-ribosylation factor (Arf) and Ras-associated binding (Rab) families, are responsible for the regulation of endosomal trafficking.

Receptor-mediated endocytosis is triggered by ligand binding to a receptor in the PM and is characterised by the formation of vesicles with the contribution of adaptor and coating proteins. In this way, endocytosis can contribute either to the maintenance of cell signalling through the recycling of receptors or to its finalisation through protein degradation. The intensity and duration of signalling would therefore be influenced by the rate of endocytosis, recycling and degradation of the receptor. Some of the receptors internalised through endocytosis are β1 and β2 adrenergic receptors and tyrosine kinase receptors (TKR) which include, among others, the epidermal growth factor receptor (EGFR), INSR, hepatocyte growth factor receptor (HGFR), vascular endothelial growth factor receptor (VEGFR), platelet-derived growth factor receptor (PDGFR), insulin-like growth factors receptors (IGFR), interleukin-2 receptor (IL2R) and dopamine 3 and 4 receptors. Interestingly, a differential signalling activity has been described for receptors depending on their location either at the PM or in specific endosomes [3].

**Figure 1:**
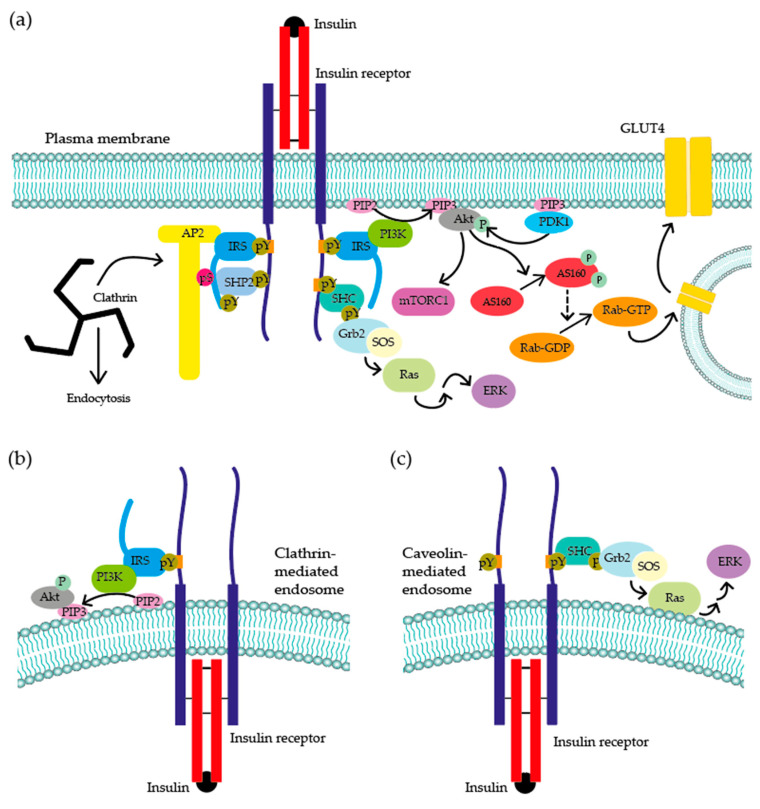
Signalling cascade initiated by insulin binding to the insulin receptor (INSR). (**a**) After insulin binding, receptors aggregate, and autophosphorylation occurs on multiple tyrosine residues, attracting the adaptor protein SHC, which becomes phosphorylated, leading to the activation of the Ras/RAF/MEK/ERK pathway. INSR phosphorylation at Y960 recruits insulin receptor substrate 1 (IRS1), phosphorylating it on Y612, Y632 and Y662. Y612 and Y632 phosphorylation are responsible for the recruitment and activation of PI3K, which leads to Akt activation and, ultimately, to GLUT4 translocation to the plasma membrane in those cells where it is expressed. IRS-1 tyrosine phosphorylation also recruits activated ERK, which causes IRS1 serine phosphorylation, attracting the phosphatase SHP2 and resulting in IRS-1 tyrosine dephosphorylation. This promotes IRS1 interaction with AP2, triggering the internalisation of INSR [9,11]. Once internalised, receptor-mediated signalling is still maintained. Indeed, in the case of clathrin-mediated endocytosis (**b**), up to 50% of insulin-stimulated PI3K activity is generated from internalised receptors [6]. However, (**c**) in cells where INSR is internalised through caveolin-1 (e.g., pancreatic β-cells), ERK activation is enhanced [7]. This indicates that, depending on the type of endocytosis and endosomes localisation, there could be separate insulin-induced activation hubs.

The insulin receptor (INSR) is a tetrameric TKR with two extracellular α-subunits and two transmembrane β-subunits linked by inter- and intra-monomer disulphide bridges. Insulin binds to the α-subunits, whilst the intracellular domain of the β-subunits contains the tyrosine kinase activity. Ligand binding to the INSR induces auto-transphosphorylation of the receptor at different residues, with the consequent recruitment of proteins such as insulin receptor substrate 1 (IRS1) as well as Ras/Raf/Mek/ERK and PI3K/Akt through downstream signalling pathway activation [4,5]. INSR localisation, either at the PM or in endosomes, has been shown to selectively activate the Ras/Raf/Mek/ERK and PI3K/Akt pathways, respectively (see Figure 1), pointing to endocytosis having a regulatory function in insulin signalling [6,7]. This review is focussed on endocytosis, receptor recycling routes and their effects on cell signalling, with a specific reference to the insulin receptor.

## 2. From the Plasma Membrane to the Early Endosome

Cells present a vast number of proteins on the surface of the plasma membrane (PM), including receptors. Some of these are regularly distributed throughout the membrane, while others concentrate in areas characterised by the presence of specific proteins on the cytosolic side. These include adaptor proteins, which interact both with membrane lipids and with the receptors, and coating proteins, such as clathrin, caveolin or endophylin, which interact with adaptor proteins and are responsible for vesicle formation. Clathrin-dependent endocytosis is considered the canonical and most frequent form of endocytic transport and is present in all cell types. Certain receptors have exclusive endocytosis mechanisms, for example, the β1 and β2 adrenergic receptors, which are only internalised by non-canonical fast endophylin-mediated endocytosis (FEME) [8]. Others, including members of the TKR family, are subject to multiple forms of endocytosis that may be dependent on the cell type. Since insulin is a key regulator of anabolic metabolism, INSRs are present in many cell types, although this receptor has been mostly studied in hepatocytes, pancreatic β-cells and adipocytes. INSR is mainly internalised by clathrin-dependent endocytosis, which has been characterised in hepatocytes, and through caveolar endocytosis, as has been described in adipocytes and pancreatic β-cells [9].

### 2.1. Clathrin-Dependent Endocytosis of INSR

Activation by insulin binding leads to autophosphorylation of INSR at multiple residues, including regulatory tyrosines 1146, 1150 and 1151 [10], which in turn results in the distribution of the receptor to clathrin-coated pits. This occurs through the anchoring of the β-subunit of INSR at an NPXY motif present on the cytoplasmic tails. Autophosphorylation at Y960 in the NPEY motif causes the direct binding of IRS1 to the INSR, which leads to INSR phosphorylating residues Y612, Y632 and Y662 of the YXXΦ motifs on IRS1. Phosphorylated Y612 and Y632 attract PI3K [11] inducing Akt activation. One of Akt substrates is AS160, a protein with a GTPase-activating protein (GAP) domain that maintains Ras-associated binding (Rab) proteins in their inactive GDP form. One of these Rab proteins, Rab10, is necessary for insulin-mediated translocation of the GLUT4 receptor to the plasma membrane. After INSR activation, active Akt phosphorylates AS160, inhibiting its GAP activity and leading to Rab10 activation (GTP-bound form) and the translocation of the glucose transporter GLUT4 in adipocytes [12].

The active form of extracellular signal-regulated kinase (ERK) is also recruited to IRS1 through phosphorylated tyrosines Y612, Y632 and Y662 and it further phosphorylates IRS1 at serines 616, 636 and 666. This serine phosphorylation provides a binding site at the C-terminal of IRS1 for SHP2, a phosphatase which dephosphorylates the tyrosine residues Y612, Y632, Y662 of IRS1 to facilitate its interaction with AP2 [9] (see Figure 1) and promotes clathrin-dependent internalisation of the receptor. The anchoring and recognition of the cargo for further sorting is regulated by the recruitment of clathrin-associated sorting proteins (CLASPs) [13], which in turn provide another binding surface for AP2 stabilization. Amongst those, LMBD1 has been suggested to be specific for INSR internalisation [14]. Following the stabilization of AP2, the β2 subunits of INSR are key in allowing for clathrin triskelion formation due to its capability to interact with clathrin through its binding domain in the β-propeller [15]. Once this cage has been assembled around the pre-vesicle, dynamin is recruited. Dynamin is a small GTPase acting as a pair of molecular scissors cutting the pinch of the invaginated region caused by the membrane curvature [16].

The process is accompanied by filament formation involving actin, which assists in the movement of the newly formed vesicle towards its next destination.

Arf6 is a member of the Arf family of small GTPases that is present in the PM and is also involved in the endocytic pathway [17,18]. Arf6 has been shown to regulate clathrin-dependent endocytosis through its GEF ARNO in insulin-induced β-pancreatic cells [19].

### 2.2. Clathrin-Independent Endocytosis of INSR

This term includes several endocytic processes that do not require clathrin coat formation, caveolar endocytosis being one of the most common. Caveolar endocytosis of INSR is found in pancreatic β-cells and adipocytes and takes place at a slower rate when compared to clathrin-mediated endocytosis. The initiation of this type of endocytosis is again triggered by the autophosphorylation of INSR, which in turn leads to the phosphorylation of tyrosine-14 in caveolin-1 [20]. In fact, the scaffolding domain present in caveolin-1 has been shown to interact with INSR [21,22].

The disruption of the actin cytoskeleton is another requirement for caveolar endocytosis [23]. This is not surprising, since this process occurs at lipid rafts rich in cholesterol and sphingolipids [24]. Therefore, ligand binding to the receptor and the consequent recruitment of caveolin-1 stimulate these microdomains to curve, thereby contributing to vesicle formation. In fact, similarities can be observed between clathrin-dependent and -independent mechanisms, since they both require coating proteins (clathrin/caveolin) to form a support to curve the membrane, so that dynamin can act to pinch off the vesicle or caveosome [16].

The physiopathological relevance of caveolar endocytosis has been demonstrated in several studies. In adipocytes, NECC2, a caveolae component that is regulated in obesity, modulates insulin signal transduction at these membrane microdomains acting as a molecular scaffold [25]. Caveolin-1 has been also involved in the regulation of voltage-dependent potassium channel Kv1.3, which participates in peripheral insulin sensitivity. Kv1.3 is targeted to caveolae by molecular interactions with caveolin-1, and its presence in caveolar raft structures is required for insulin signalling [26]. These examples highlight a differential effect on the activation of INSR, merely for the co-localisation with critical signalling partners restricted to that compartment. Furthermore, lipidic composition and fluidity of the PM are also relevant factors in caveolar endocytosis. For example, an excess of sphingomyelin phosphodiesterase acid-like 3b (SMPDL3b), a lipid raft enzyme that regulates PM fluidity, has been shown to prevent insulin signalling by interfering with INSR binding to caveolin-1 in the PM [27].

### 2.3. Uncoating and Binding to Early Endosome

In clathrin-mediated endocytosis, two steps are required: first, coating and then de-coating the vesicle. The former is needed to assist vesicle formation from the membrane, and the latter to prepare the vesicle for its proper fusion with the early endosome. Current models for uncoating point to the binding of auxilin to the assembled clathrin triskelion as being responsible for guiding the recruitment of ATP-activated chaperon Hsc70 via its J-domain. This will then stimulate the ATPase activity of the chaperones, leading to the conformational unfolding and disruption of clathrin molecules and, eventually, to the release of the clathrin sheet [28,29,30]. Following the release of clathrin, AP2 is also released due to the recruitment of hRME-6, which displaces AP2-associated kinase 1 (AAK1), causing µ2 dephosphorylation of the AP2 protein. Additionally, the small GTPase Rab5 controls the turnover of PI (4,5)P2, contributing to the release of AP2 from the vesicle, so the fusion of membranes can take place [31].

## 3. Sorting at the Early Endosome

The early endosome (EE) is known as the major sorting compartment within cells, controlling the trafficking of internalised cargo to be appropriately compartmentalised, either towards recycling or to the lysosomal path for degradation [32]. The continuous maturation of the EE gives rise to multivesicular bodies (MVB), vesicles capable of transporting receptor–ligand complexes, nutrients, membrane proteins, etc., to the late endosome or back to the membrane. The EE is a dynamic organelle with microdomains that contribute to the distribution of the cargo and is characterised by a specific membrane protein profile. Among those, are the already mentioned Rab family, small GTPases that can be prenylated in the C terminus region and localise to membranes. In fact, three-quarters of all human Rab GTPases are involved in endosomal trafficking, coordinating the various protein interactions involved in these pathways. Another family of small GTPases, Arf, present in the PM and in endosomes in a myristoylated form, are also involved in the regulation of endosomal trafficking. Both GTPases are activated by guanine exchange factors (GEF) and inactivated by GTPase-activating proteins (GAP). Regarding their effector molecules, Rab GTPases can interact with coat, motor, tethering and SNARE proteins; Arf GTPases interact with coat proteins and adaptors. Among their other functions, members of both families control vesicle transport and membrane trafficking in the endocytic and exocytic systems and have been shown to be involved in many physiological and pathological processes [33,34].

In the EE, there are two Rab family members that have been extensively studied, Rab5 and Rab4, although others including Rab10, Rab14, Rab21 and Rab22 have been also described. Rab5 is activated when bound to GTP and requires stabilisation and to be present at elevated levels to recruit its effectors. The first effector protein thought to be recruited by Rab5 is class III phosphatidylinositol 3-kinase VPS34 which, together with GTP-Rab5, can then recruit EEA1, rabenosyn-5 and sorting nexins (SNX). SNX are members of an extensive family of more than 30 proteins which play key roles in endocytic trafficking, including endocytosis, endosomal sorting and endosomal signalling. EEA1 functions with members of the SNARE family to facilitate the fusion of vesicles. Rabenosyn-5, with its versatile FYVE-domain that can interact with both Rab4/5, is responsible for the coordination of sorting processes and fast recycling of the receptors directly back to the plasma membrane. APPL1 and APPL2 (adaptor protein containing PH domain, PTB domain and leucine zipper motif) are Rab5 effectors involved in insulin signalling pathways and considered important mediators of insulin sensitisation [35]. Indeed, these proteins are present in very early endosomes, before EEA1 recruitment [36,37], acting as sites of cargo sorting [38]. In addition, they have a role in signalling, regulating, for example, Akt activity and IRS1/2 activation [39].

### 3.1. Sorting for Degradation

The fate of the cargo at the EE depends on the sorting signals present in the membrane. Sorting for the degradation pathway requires the mono-ubiquitination of lysine residues in the tyrosine kinase region of TKRs, including INSR [40]. Depending on the cell type, slightly different mechanisms are involved. In hepatocytes, the recognition of the ubiquitinated receptor is via hepatocyte growth factor-regulated tyrosine kinase substrate (Hrs), through a ubiquitin-interacting motif (UIM) [41]. Binding of Hrs leads to the formation of a specialised sorting microdomain which guides the cargo for degradation and away from tubular-forming recycling vesicles. Other proteins possessing the UIM domain, including Eps15 and signal transducing adaptor molecule 2 (STAM2), expedite the interaction with ubiquitinated cargo for stabilization. The EE cargo must then be transported through invaginations of the membrane, leading to the formation of MVB, which later merges with the lysosomal vesicles. The Hrs protein capacity to attach to the Tsg101 subunit of ESCRT-I (Endosomal Sorting Complex Required for Transport), contributes to the recruitment of the ubiquitinated cargo towards the late endosomal compartment [42,43].

### 3.2. Sorting for Recycling

**Figure 2:**
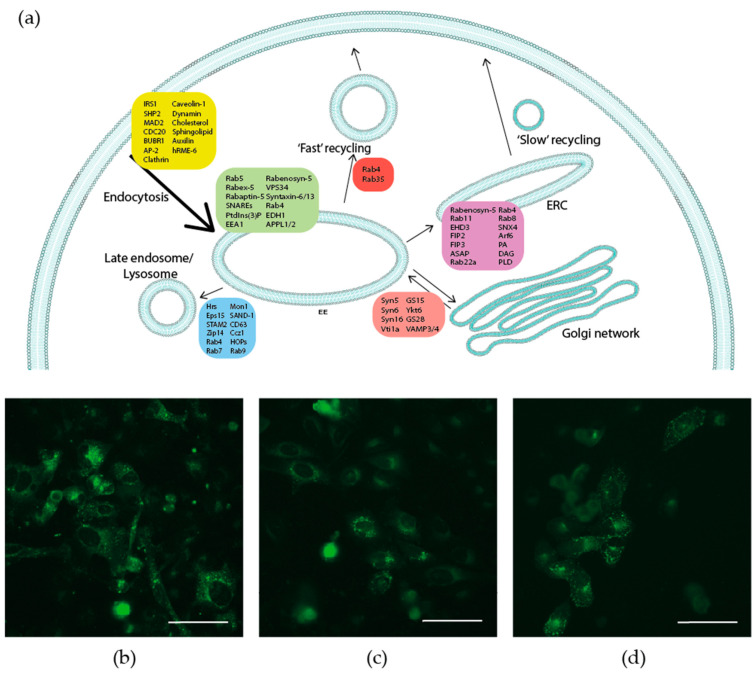
Distribution of endosomal compartments. (**a**) Diagram representing the different endosomal compartments and the proteins that can be found in them (EE, early endosome; ERC, endosomal recycling compartment). Below, human osteosarcoma U2OS cells transfected with GFP-tagged versions of (**b**) Rab5B, a gift from Gia Voeltz (Addgene plasmid #61802 [44]); (**c**) Rab7; and (**d**) Rab11, both gifts from Richard Pagano (Addgene plasmid #12605 and #12674, respectively [45]). Stably transfected cells were selected in the presence of gentamycin (500 µg/mL), and the cellular localisation of the different Rab isoforms was visualised with an EVOS FLoid microscope. Bar: 100 µm.

Little is known about the specific mechanisms involved in INSR sorting for recycling and in the recycling process itself. However, the information gathered on other receptors provides a general reference model for this process. Recycling is fully dependent on the level of tubulation of the EE membrane. Two main forms of recycling have been described so far, one termed ‘fast recycling’, responsible for the direct route from the EE to the PM, and a slower recycling route that organizes cargo for transport to the endocytic recycling compartment (ERC), a region of endosomes near the nucleus (see Figure 2 for an overall diagram). 

Sorting of the cargo for recycling has been shown to be regulated mainly by Rab proteins 4 and 11. It is believed that Rab4, along with geometry-based sorting, leads the cargo to the fast route, directing it into newly formed tubular membranes. In the slow recycling route, the cargo must first go to the ERC before eventually reaching the PM in a process controlled by Rab11 [46]. Arf 1 and 6 may cooperate with Rab11 to control this process [47,48]. Rab4 shares some effector proteins with Rab5, which contributes to vesicle formation from the EE. An essential effector protein that regulates the delivery of the cargo to the ERC is rabenosyn-5, since its depletion leads to the accumulation of the cargo in the EE. Rabenosyn-5s also binds to EDH1, regulating perinuclear activity including events that take place at the ERC [49].

Besides these well-known pathways, recycling as a result of recognition of sorting domains in cargo molecules has also been described at the EE. This is a regulated process involving actin, which has a key role in both sorting receptors into tubules and maintaining tubules integrity [50], a Wiskott–Aldrich syndrome and SCAR homologue (WASH) complex retromer/retriever; and SNXs (comprehensively reviewed in [51]). Interestingly, signalling molecules such as the β2-adrenergic receptor (B2AR) and other G protein-coupled receptors can be recycled from these actin/sorting nexin/retromer tubular (ASRT) microdomains. In the case of B2AR, phosphorylation of its S345 and S346 by PKA targeted the receptor to ASRT domains. This sequence-dependent sorting had further consequences, as B2AR initiated G protein-mediated signalling from ASRT domains only [52], highlighting a specific receptor-mediated signalling role for an endosomal compartment.

## 4. From the Late Endosome to the Lysosome

The transformation of EE into late endosomes (LE) has been described either as a transport process, involving endosomal carrier vesicle (ECVs) and/or multivesicular bodies (MVBs) formation [53], or as a maturation process in which the endosome itself changes before merging with lysosomes. In fact, LE share several features with lysosomes, including an acidic pH and the capacity to sense nutrients through the mTOR pathway [54], and have been suggested to form an endolysosomal system aimed at the degradation of cargo [55]. Transport or maturation into LE is controlled by changes in the presence and activity of different members of the Rab family, the most relevant of those being Rab5 substitution by Rab7 [56]. This event occurs at the EE and may involve the splitting into MVB, a microtubule-dependent process in which the Rab7 region dissociates from the Rab4/Rab5 endosomal compartment. The recruitment of the Rab7 GEF Mon1/SAND-1 complexed with Ccz1 dissociates rabex-5 from the Rab5 complex, leading to the inactivation of Rab5, its departure and the disassembly of the complex. This provides the Mon1/SAND-1–Ccz1 complex with an interacting site for the recruitment and activation of Rab7 as well as members of the HOPS family, which assist in membrane tethering and fusion [57]. The key role played by Rab7a in receptor degradation and cell signalling regulation has been demonstrated in β-pancreatic cells under metabolic stress. Inhibition of Rab7 increased IGF-I receptor density and signalling and promoted β-cell survival, suggesting this could be a therapeutical approach for type-II diabetes [58].

## 5. The Recycling Endosome

While fast recycling happens directly from the EE, slow recycling involves the ERC, a perinuclear group of endosomes which are related to the Golgi apparatus and the endoplasmic reticule (ER). The ERC presents Rab11 as a characteristic marker together with Rab8, Rab22a and Arf6. Some proteins have been shown to be key regulators of ERC trafficking. EH domain (EHD)-containing proteins coordinate, among other functions, recycling through interactions with the Rab11 pathway through its effector protein Rab11-FIP2 [59]. EHD can also bind to the NPF (Asn–Pro–Phe) motif of the Rab5 effector rabenosyn5, suggesting its role as a linker protein between the EE and the ERC [60]. Some SNXs are responsible for preventing entry to the degradation route and for leading the vesicles towards the ERC. For example, SNX4 operates by binding to the dynein motor through a KIBRA linker protein, facilitating its movement away from the late endosome pathway [61].

Arf6 plays a relevant role in the recycling from the ERC to the PM. The interaction of Arf6 effector scaffold proteins FIP3 and FIP4 with either dynein or kinesin determines the vesicle direction, either to the ERC or from the ERC to the PM. Phosphorylation of FIP3 works as a molecular motor switch, inducing the change of dynein for kinesin 1, leading to transport towards the PM [18]. Arf6 has also been shown to activate phospholipase D, which is expressed on tubular recycling membranes. The lipid products of phospholipase D phosphatidic acid (PA) and DAG are both responsible for the action of recycling carriers, where PA impacts on proteins related to the fission and release from the ERC, and DAG influences the fusion of the carriers to the plasma membrane. Finally, Arf6 activates phosphatidylinositol 4-phosphate 5-kinase (PIP5-kinase), with the subsequent production of PtdIns(4,5)P2 (PIP2), a key step in endocytosis and endosomal recycling [18].

## 6. Perinuclear ERC—A Means to Target Proliferation and Migration in One Go?

As mentioned before, it has been proposed that the role of the signalling cascade generated by receptor activation can differ depending on the cellular compartment the receptor is located. In the case of insulin, within 2–5 min of administration, the INSR internalised in endosomes presents its maximal activity, higher than the activity achieved at the plasma membrane [62]. It is therefore not surprising that, as INSR activation results in the stimulation of PI3K–Akt signalling, PI3K has also been found targeted to these compartments [63]. In practice, all the machinery necessary to generate the required signalling cascade seems to be in place. Subsequently, the disruption of the normal endosomal function has been proposed as one of the causes of impaired insulin signalling in the brain [64]. Therefore, with the efficiency that characterises cellular processes, the existence of a fast and a slow route for the recycling of receptors to the membrane ought to serve specific functions, and clues about their role may lie in their localisation.

The perinuclear ERC is located, in most cells, in the perinuclear region adjacent to the microtubule-organising centre (MTOC). The MTOC contains the centrosome, key for cell division, where microtubules are nucleated. In addition, microtubule nucleation can happen directly at the Golgi membrane, supporting Golgi homeostasis and a balanced exchange with endocytic membranes, as well as directional cell migration and possibly cell division [65]. The Golgi apparatus is also located close to the MTOC, and these structures affect each other’s positioning. Furthermore, they both serve as actin nucleation centres [66], which brings them into the realm of intracellular transport regulation and cell migration, as intracellular vesicles move along microtubules, and cell movement is dependent on the actin cytoskeleton. The ERC being in such proximity to the Golgi and the MTOC offers a unique opportunity for the slowly recycled receptors to regulate the functions carried out by these structures. For example, insulin is well known to facilitate proliferation and migration through the activation of the PI3K–Akt pathway, and the localisation of INSR within the REC would bring active Akt to the surroundings of the MTOC and the Golgi.

### 6.1. Role of ERC in Cell Cycle and Proliferation

As already mentioned, Rab11 was identified as the first small GTPase associated to the recycling endosomes [67], also helping in the exocytosis of these vesicles at the plasma membrane [68]. There is growing evidence of a close relationship between Rab11, the centrosome and the control of the cell cycle. For example, Rab11 binds Evi5, a protein that accumulates in early G1 phase and helps the G1–S transition by stabilising the anaphase-promoting complex/cyclosome inhibitor Emi1 [69]. Evi5 activity is regulated by protein stability, and its degradation can be triggered by the Polo-like kinase Plk-1 [70]. Interestingly, insulin-mediated signalling can regulate Plk-1 expression by increasing the DNA-binding activity of the transcription factor FoxM1, which leads to *PLK1* transcription [71], promoting mitosis.

During mitosis, the spindle fibres grow from the centrosome, and Rab11 has been shown to associate with the centriole through binding to cenexin [72] (the overall relevance of recycling endosomes in centrosome biology, function and duplication has been recently summarised [73]). Interestingly, Rab11 is also implicated in the completion of cell division, being required for successful cytokinesis [74], in a process that involves Fyn-mediated signalling [75]. In this regard, the Src kinase Fyn has been reported to bind IRS1 after insulin stimulation [76]. Therefore, a recycled INSR that is still active in the ERC could attract Fyn and contribute to cytokinesis, favouring the successful completion of cell division.

### 6.2. Role of ERC in Cell Migration

Cells depend on their cytoskeleton for successful migration. Cell movement is a coordinated event and, as mentioned earlier, the ERC is located beside the Golgi and the centrosome, which are actin nucleation centres [66]. Indeed, Golgi-derived microtubules were able to control the speed of cell migration during wound closure [77]. There is growing evidence of the participation of Rab11 in cell migration, with several Rab11 family-interacting proteins (Rab11-FIP2 and Rab11-FIP4) involved in cancer cell migration [78,79,80] and Rab11 itself involved in collective cell movements [81]. In terms of the involvement of TRKs in ERC-mediated migration, there is not much evidence. However, IGF-I, which together with insulin is one of the ligands that bind to INSR, has been shown to induce cell migration in an IRS1- and IRS2-dependent manner [82]. IGFR can be located at the Golgi, and therefore at the perinuclear area, where it was shown to play an active role in regulating cell migration [83,84]. In addition, high levels of glucose and insulin, characteristic of diabetes, induce cell proliferation and invasion by upregulating IRS1 and activating the Ras/Raf/ERK pathway [85]. It would be interesting to check if the levels or activity of Rab11 or any of its interacting proteins can be regulated by IRS1.

## 7. Perspectives

Endosomal trafficking is an area of active research. How the cargo is sorted, what the exact relationship between organelles and the process of autophagy (not covered here) is and how these movements are orchestrated is still under investigation. New players are continually being discovered, and recent evidence supplies examples of specific signalling regulation in different compartments.

Our group, has been working on the Arf GAP protein AGAP2, a ubiquitous protein that stabilises Akt in its active conformation. AGAP2 can interact with the clathrin adaptor protein AP1 and has also been shown to form complexes with β-arrestin1 and β-arrestin2, participating in the recycling of B2AR to the plasma membrane. We observed a similar phenomenon with other receptors: AGAP2 knockdown prevented the recycling of TGFβRs to the membrane, leaving the receptors in the perinucleus [86] and the same pattern was observed with INSR (unpublished data). These results suggest that AGAP2 has a role in receptor trafficking that may be wider than originally thought. Moreover, we also found that the impairment of AGAP2 activity leads to a reduction in cell migration induced either by TGF-β or by insulin. Suddenly, the potential for recycling endosomes to be an efficient way of controlling receptor signalling and their effects on cell proliferation and/or migration seems highly plausible. Further studies will be required to establish the specific contributions of regulator proteins such as Rab, Arf and their GAPs and GEFs in the complex net of signalling events involved in endocytosis and endosomal trafficking. The fact these pathways are altered in different pathologies also opens a promising field for the design of new therapies.
